# Comparison of Diagnostic Cytomorphology of Natural Killer/T-Cell Lymphoma (Nasal Type) in Conventional Smears, Liquid-Based Preparations, and Histopathology

**DOI:** 10.1155/2018/6264810

**Published:** 2018-05-14

**Authors:** Chih-Yi Liu, Hui-Chih Tsai

**Affiliations:** ^1^Division of Pathology, Sijhih Cathay General Hospital, New Taipei City, Taiwan; ^2^College of Medicine, Fu Jen Catholic University, New Taipei City, Taiwan

## Abstract

Natural killer (NK)/T-cell lymphoma is formally referred to as extranodal NK/T-cell lymphoma, nasal type (ENKTCL), in the 2008 and 2016 World Health Organization (WHO) classifications. NK/T-cell lymphoma, nasal type, is a rare but clinically important lymphoid neoplasm. It is the predominant type of extranodal lymphoma associated with the Epstein–Barr virus (EBV). NK/T-cell lymphoma is marked by a wide cytomorphological spectrum. The cytological findings may be so subtle that NK/T-cell lymphoma could possibly be easily overlooked. Here, we report a case of NK/T-cell lymphoma involving the sinonasal region with lymph node involvement. Fine needle aspiration of the neck lymph node and punch biopsy of the nasal mucosa were performed. The diagnosis of NK/T-cell lymphoma was confirmed based on pathological and immunohistochemical analyses, as well as in situ hybridization for EBV-encoded mRNA (EBER). The present case report underlines the importance of prompt clinicopathological assessment in suspected cases. The comparison of cytomorphologic features of NK/T-cell lymphoma in various specimens is presented.

## 1. Introduction

Extranodal NK/T-cell lymphoma, nasal type (ENKTCL), is a unique form of mature NK/T-cell lymphoma that is invariably associated with the Epstein–Barr virus (EBV) and exhibits a predilection for occurrence in certain ethnic groups. The disease is more prevalent among Asians and Native Americans as compared with the populations in the western countries [1–3]. In the majority of cases, this disease occurs in the nasal cavity and other parts of the upper aerodigestive tracts. However, such “nasal-type” tumors can also be encountered at other anatomic sites. The clinical course is typically aggressive, and it is associated with high mortality [1–4]. Herein, we present a case of NK/T-cell lymphoma in which the diagnosis was established based on examination of both cytological and histological specimens. The key clinical and cytopathological features of this rare hematolymphoid disorder are also reviewed.

## 2. Case Report

**Figure 1:**
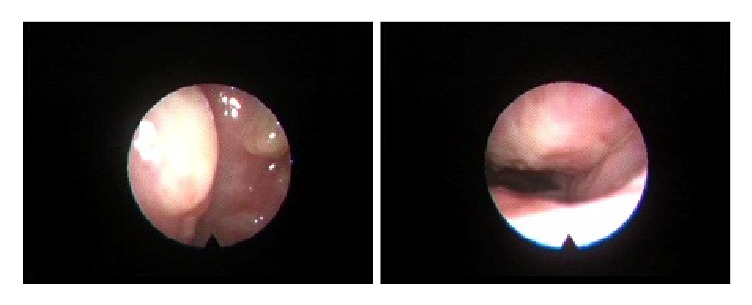
Fiberoptic endoscopic examination of the left nasal cavity shows diffusely whitish exudates coating on the mucosal surface.

**Figure 2:**
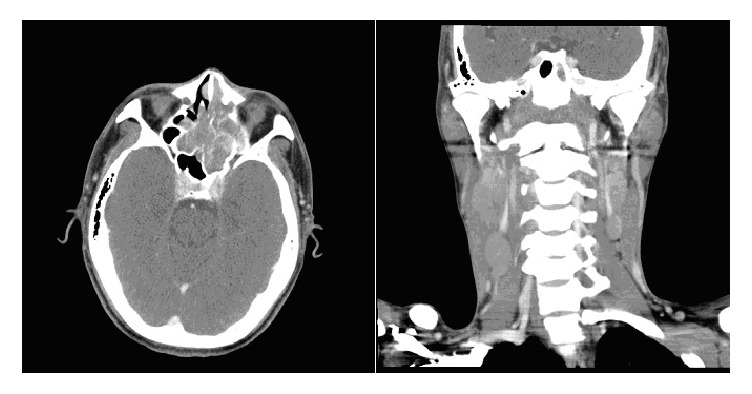
Contrast-enhanced CT of the head and neck reveals iso-enhancing masses in the left nasal cavity, nasopharynx, and paranasal sinuses, accompanied by bilateral neck lymphadenopathy.

A 39-year-old man presented with nasal obstruction accompanied with foul-smelling discharge for the last 1 month. In addition, he complained of headache, poor appetite, and bilateral palpable neck masses. On physical examination, firm and nontender lymph nodes were found over bilateral level II regions. Fiberoptic endoscopy of the left nasal cavity showed mucosal erosions and whitish exudates coating the mucosal surface (Figure 1). Laboratory examination revealed mild anemia and elevated serum lactate dehydrogenase level. HIV test result was negative. Computed tomography of the head and neck demonstrated iso-enhancing masses in the left nasal cavity, nasopharynx, and paranasal sinuses associated with destruction of the adjacent bony structures. Multiple enlarged and enhancing lymph nodes were observed at bilateral levels IB, II, III, IV, and V (Figure 2). Based on the clinical suspicion of malignancy, fine needle aspiration (FNA) of the neck lymph node and punch biopsy of the nasal mucosa were performed.

Ultrasound-guided FNA cytology (FNAC) was performed by standard technique using a 22-gauge needle for the left neck lymphadenopathy. One air-dried and one alcohol-fixed smear, as well as liquid-based cytology (LBC, SurePath) specimen, were prepared for cytological evaluation. The cell sample was fixed in CytoRich fixative, and additional cell block was also prepared from the remnants of LBC specimen. The air-dried smear was stained with Liu's stain. The alcohol-fixed smear was stained with Papanicolaou stain.

**Figure 3:**
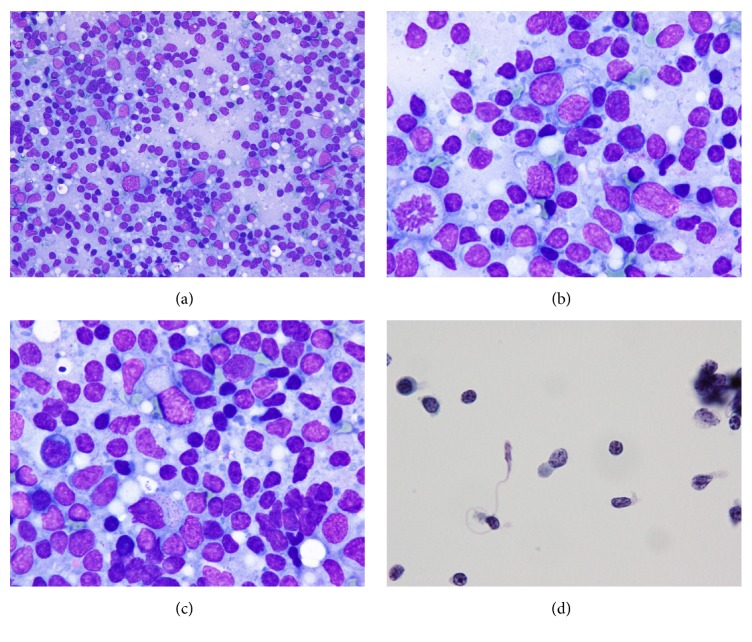
FNAC of the left neck lymph node. (a) Lymphoid cells varying in size and shape (Liu's stain, 400x). (b, c) Intermediate to large cells with irregular nuclei, central nucleoli, tongue-like cytoplasmic protrusions, and fine or coarse azurophilic granules (Liu's stain, 1000x). (d) Cytoplasmic protrusions are visible in LBC (Pap stain, 1000x).

**Figure 4:**
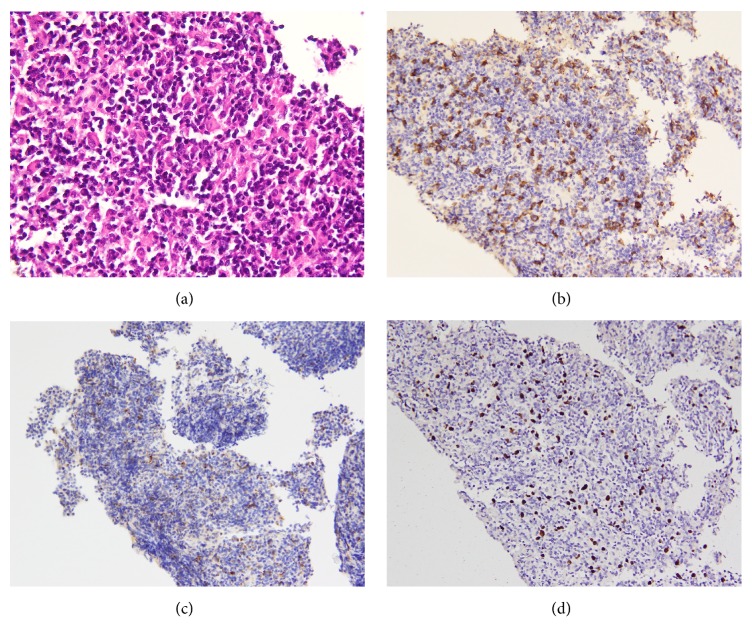
Cell block preparation from the residual FNAC specimen. (a) Neoplastic lymphoid cells with nuclear irregularity (H&E stain, 400x). Immunocytochemical analysis shows lymphoma cells positive for CD56 (b) and granzyme B (c). Nuclei are positive for EBER by in situ hybridization (d) (200x).

FNAC revealed dispersed hypercellular pattern, with a polymorphous mixed population of small-, intermediate-, and large-sized lymphoid cells. Follicular center cells, histiocytes (including tingible body macrophages), and lymphohistiocytic aggregates were not found. Plasma cells and eosinophils were scant in numbers. In Liu's stain slide, lymphogranular bodies were well recognized in the background. There were medium-to-large atypical cells, which exhibited irregular nuclei with one to several nucleoli and pale blue or clear cytoplasm. Cytoplasmic projections with tongue-like protrusions and fine or coarse cytoplasmic azurophilic granules were also present (Figure 3). Mitotic figures were readily found. Necrosis or karyorrhexis was inconspicuous. In LBC specimen, the atypical lymphoid cells were oval or elongated in shape. Variously shaped cytoplasmic processes were also visible in Papanicolaou stained slides (Figure 3). Cell block section revealed scattered medium- to large-sized lymphoid cells with pleomorphic nuclei and small nucleoli among a mixed population of small lymphocytes, plasma cells, and histiocytes. The immunophenotype of atypical cells was thus demonstrated by cell block sections (Figure 4).

**Figure 5:**
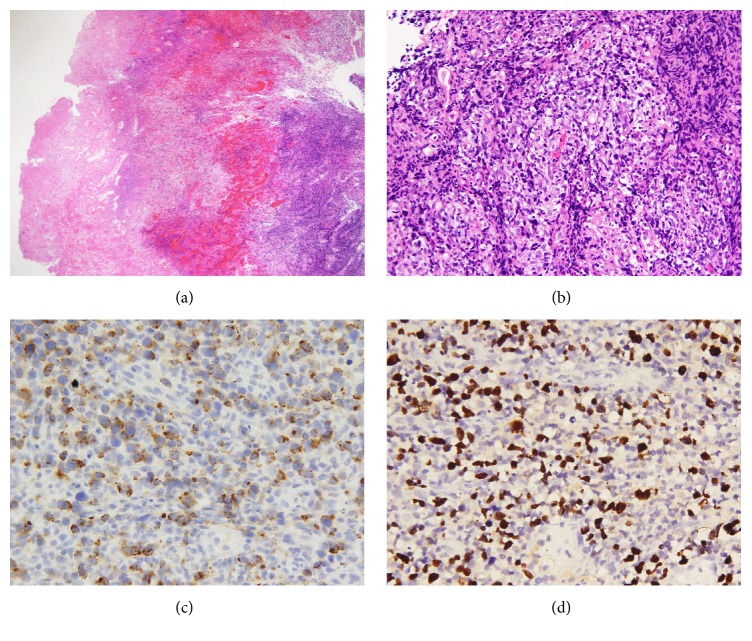
Morphological features of nasal tumor. (a) Low-power view shows diffuse cellular infiltrates with necrosis and mucosal ulcer (H&E, 100x). (b) High-power view shows a mixture of small-, medium-, and large-sized cells with angiocentric growth pattern (H&E, 200x). Immunohistochemical study: (c) the tumor cells are positive for granzyme B (C). Nuclei are positive for EBER by in situ hybridization (d) (400x).

The biopsy specimen of the left nasal lesion showed diffuse mucosal necrosis with the presence of granulation tissue and dense cellular infiltrates. Atypical lymphoid cells of varying sizes with prominent nuclear irregularity and pale or clear cytoplasm were also observed. Large atypical lymphoid cells, significant apoptosis, and scattered mitotic figures were noticed. The neoplastic cells exhibited an angiocentric growth pattern, as well as angiodestruction with mural fibrinoid necrosis and hemorrhage (Figure 5). Pseudoepitheliomatous hyperplasia of residual squamous epithelium was also observed. On immunohistochemical staining, the atypical lymphoid cells stained positive for CD56, granzyme B, and CD3 and negative for CD20 and cytokeratin (AE1/AE3) (Figure 5). Most of the atypical lymphoid cells exhibited CD4−/CD8− phenotype. Mixed CD4+ or CD8+ small lymphocytes were observed in the background. The neoplastic cells tested positive on in situ hybridization for EBER (Figure 5). The diagnosis of NK/T-cell lymphoma, nasal type, was confirmed based on the histopathological features and the results of the ancillary studies. The patient was subsequently hospitalized and given sequential chemotherapy and radiotherapy for three months.

## 3. Discussion

Natural killer (NK)/T-cell lymphoma is formally referred to as extranodal NK/T-cell lymphoma, nasal type (ENKTCL), in the 2008 and 2016 World Health Organization (WHO) classifications. NK/T-cell lymphoma located in the nasal region is the prototype for this lymphoma, which was formerly referred to as polymorphic reticulosis. The disease typically affects the nose and facial midline and exhibits aggressive destruction caused by cytotoxic activity, due to which it was classified as a lethal midline granuloma [1–4]. NK/T-cell lymphoma, nasal type, is associated with a wide spectrum of histopathological changes. The most common finding is that of a polymorphous infiltration of small, medium, and large atypical lymphocytes interspersed with plasma cells, macrophages, and neutrophils. NK/T-cell lymphoma, nasal type, was formerly designated as an angiocentric lymphoma owing to common findings of necrosis and angiocentric infiltration or angioinvasion [2, 3]. Accurate diagnosis of NK/T-cell lymphoma is a clinical challenge, especially based on small biopsy specimens as the neoplastic cells are often admixed with inflammatory cells and necrosis. The neoplastic cells may consist only of small lymphocytes with no obvious atypia and/or necrosis, which are liable to be overlooked and lead to a misdiagnosis of chronic inflammation [3, 4].

The immunophenotype of typical NK/T-cell lymphoma is marked by positive staining for CD2, CD56, and cytoplasmic CD3*ε* and negative staining for surface CD3. Lymphoma cells exhibit positive expression of cytotoxic granule proteins such as granzyme B, TIA-1, and perforin. Other markers such as CD43 and CD45RO may be positive; positive expression of CD30 may also be found in some cases [1–4]. While LMP-1 immunoreactivity may vary, virtually all lymphoma cells should be labeled with EBER on in situ hybridization. The TCR and Ig genes are in a germline configuration in the majority of cases [1–5]. A small proportion of cases show rearrangements of the TCR genes (up to 27%), which probably represent neoplasms of cytotoxic T-cells [3, 6].

To the best of our knowledge, the cytological features of NK/T-cell lymphoma, nasal type, are not well established in the published literature [7–14]. NK/T-cell lymphoma is marked by a wide cytological spectrum, which varies from a predominance of small cells to a predominance of large cells. On FNA smears, a mixture of benign-appearing small lymphocytes and variable numbers of small to large atypical lymphoid cells is usually seen [7–14]. Plasma cells and, less often, eosinophils and histiocytes are also present. The appearance of the neoplastic population tends to vary in individual cases. In most cases, medium-sized cells with granular chromatin; multiple small, inconspicuous nucleoli; and pale cytoplasm are observed. The tumor cells show readily discernible nuclear atypia with an irregular nuclear outline (including elongated, twisted, “banana shaped,” or “cucumber-like” cells) [10]. The presence of cytoplasmic “tongue-like” protrusions may serve as a useful diagnostic clue [11, 12]. Cytoplasmic azurophilic granules are often identified in touch preparations of the tissue samples [7, 9, 11, 12, 14]. Abundant necrotic areas and karyorrhectic debris may be present.

Cytological diagnosis based on FNAC specimens is typically straightforward in case of certain subtypes of lymphoma, such as the conventional diffuse large B-cell lymphoma and Burkitt's lymphoma, which are marked by a monotonous tumor cell population [10]. In contrast, a false-negative cytological diagnosis is a distinct possibility in the case of lymphomas that consist of a heterogeneous cell population; in such cases, the lymphoma cells may be masked by the background reactive hematolymphoid cells. A heterogeneous distribution and a polymorphic lymphocytic population, with cellular aggregates representative of germinal centers, usually point toward nonspecific reactive follicular hyperplasia. Nevertheless, reactive hyperplasia is typically marked by a predominance of large cells and immunoblasts, sometimes even with atypical forms (common in clinical conditions associated with viral infections, such as infectious mononucleosis). Immunostaining and flow cytometry may be employed for further characterization of such cases [10, 13].

Cytological differential diagnosis of NK/T-cell lymphoma from other types of lymphoma is a challenge. Diffuse large B-cell lymphoma typically exhibits large cells with rounded noncleaved nuclei. Grade 1 or grade 2 follicular lymphoma shows predominantly medium-sized centrocytes with irregularly cleaved nuclei, centroblasts with noncleaved nuclei, and follicular aggregates on Pap staining. In contrast to the Reed–Sternberg cells in Hodgkin's lymphoma, the atypical lymphoid cells of NK/T-cell lymphoma do not contain large, inclusion-like, eosinophilic nucleoli [10, 13]. NK/T-cell lymphoma displays a wide cytological spectrum, from small to large pleomorphic cells, and a necrotic background containing abundant apoptotic material. NK/T-cell lymphomas are also marked by morphological diversity and a broad cytological spectrum. There is morphological overlap among these tumors. Thus, a combination of immunophenotypic studies and molecular biological studies is essential for the differential diagnosis of T-cell and NK cell lymphomas. The characteristics of the major differential diagnoses are listed in Table 1.

In the current case, FNAC of the lymph node shows a heterogeneous population of small-, medium-, and large-sized lymphoid cells, with the lack of reactive cellular components such as follicular center cells or lymphohistiocytic aggregates. The medium- to large-sized lymphoid cells exhibit readily discernible nuclear atypia with an irregular nuclear outline. The cellular shrinkage is obvious in LBC specimen, while the cytoplasmic protrusion is more easily visible than conventional smears. The degree of nuclear atypia far exceeds that of reactive changes. Lymphoma cells with elongated and twisted nuclei, recapitulating the cellular morphology seen in biopsies of the usual nasal-type NK/T-cell lymphoma, are common [10]. The NK/T-cell lymphoma cells show similar features in the previous case reports, including rich and pale blue cytoplasm with azurophilic granules and characteristic tongue-like cytoplasmic protrusions [7–14]. The azurophilic granules are easily recognized in Giemsa stain [11]. The above features are demonstrated in our case, and these intriguing cytologic findings are indicators of NK/T-cell lymphoma. Thus, a combination of medium- to large-sized atypical lymphoid cells with irregularly twisted nuclei, together with an absence of reactive cellular populations, should at least raise a suspicion of T-cell lymphomas or NK/T-cell lymphoma in lymph node fine needle aspirates. In the present case, cell block preparation from residual LBC materials is an important adjunct to the diagnostic interpretation. The characteristics of neoplastic cells are less conspicuous but rather preserved in LBC specimen. Besides, immunocytochemical study, including ISH for EBER, can be readily applied to cell block paraffin sections. Correct cytological assessment could be reached based on careful interpretation of the cellular features and the ancillary techniques [15].

In a recent case series of NK/T-cell lymphoma, nasal type, in Taiwan, a total of 73 cases were retrospectively investigated with a median age of 54 years and a male-to-female ratio of 2.0 : 1 [16]. The upper aerodigestive tract (nasal group) was the most common site of involvement (70%). The other organs included the skin (16%) and gastrointestinal tract (7%). Excluding those with indeterminate lineage, 75% of the cases were of NK lineage and 25% were of T lineage. Extranasal tumors were more aggressive than their nasal counterparts [16]. On the other hand, Kuo et al. further classified nasal ENKTCL into small cell, medium-sized cell, large cell and pleomorphic cell types [17]. Notably, the small cell type did not show vascular destruction, thrombosis, or necrosis, although vascular infiltration was occasionally seen. Three cases of other histologic subtypes did not have tissue necrosis. Since nasal biopsy specimens were usually small, some of the variations in microscopic findings might partly be attributable to sampling variation [17]. Thus, correct cytologic assessment of NK/T-cell lymphoma relies heavily on careful interpretation of cellular features and their patterns as well as on a high index of suspicion.

Although ENKTCL is sensitive to radiotherapy, it shows a poorer response to chemotherapeutic agents than other lymphomas due to expression of p-glycoprotein [18]. At present, radiotherapy followed by chemotherapy is regarded as a standard strategy for limited stage ENKTCL. The prognosis of advanced stage ENKTCL, nasal type, is extremely poor when using any chemotherapeutic regimen [18]. Early and correct pathological diagnosis and prompt treatment of NK/T-cell lymphoma are important owing to its typically aggressive behavior.

Definitive cytomorphologic diagnosis of lymphoma is possible but can be challenging in routine cytology specimens. Review of a previous case series report showed that a diagnosis of NK/T-cell lymphoma is possible on FNA or body fluid cytology, given adequate ancillary testing support. Cell block material, if available, is an important adjunct to diagnosis.

Immunocytochemical study, as well as more sophisticated techniques, can be readily applied to cell block paraffin sections. Consideration of clinical features, cytology, and/or histomorphology and determination of immunophenotype on immunohistochemical examination or flow cytometry are useful for arriving at a definitive diagnosis.

## Figures and Tables

**Table 1:** Summary of major differential diagnoses of NK/T-cell lymphoma, nasal type.

	NK/T-cell lymphoma, nasal type	Reactive lymphoid hyperplasia	Follicular lymphoma, G1/2
Cellular composition	Heterogeneous lymphoid population of small, intermediate and large-sized lymphoid cells in varying proportions	Heterogeneous lymphoid population with small lymphocytes predominating large lymphoid cells	Monotonous, small to intermediate-sized lymphoid cells

Nuclear features	Irregularly folded or elongated nuclei Dense granular chromatin and inconspicuous or small nucleoli	Small, round to oval, non-cleaved or angulated nuclei Evenly dispersed chromatin Smooth nuclear membrane and inconspicuous nucleoli	Irregular nuclear outlines, cleaved, notched or with grooves Paler and evenly dispersed chromatin Smooth nuclear membrane and inconspicuous or one to several small nucleoli

Cytoplasmic features	Moderate amounts of pale to clear cytoplasm Cytoplasmic projections with tongue-like protrusion Azurophilic granules (Giemsa-stain)	Scant or little cytoplasm Cytoplasmic projection (−) Azurophilic granule (−)	Scant and pale cytoplasm in centrocytes Cytoplasmic projection (−) Azurophilic granule (−)

Background features	Tingible body macrophages (−) Dendritic/histiocytic cells (−) Lymphohistiocytic aggregates (−) Admixture of inflammatory cells (+/−) Necrotic background (+/−)	Tingible body macrophages (+) Dendritic/histiocytic cells (+) Lymphohistiocytic aggregates (+/−) Admixture of inflammatory cells (+/−) Necrotic background (−)	Tingible body macrophages (rare) Dendritic/histiocytic cells (rare) Lymphohistiocytic aggregates (−) Admixture of inflammatory cells (−) Necrotic background (−)

IHC study	Positive: CD2+, CD3, CD56, CD43+, CD45RO+, granzyme-B, TIA-1 Negative: CD4, CD8, CD20 EBER: Positive	Polyclonal B-cells (CD20+) and T-cells (CD3+) Absence of CD10+/Bcl-2+ co-expression in follicular B-cells EBER: Negative	Positive: CD19, CD20, CD79a, CD10, Bcl-2, Bcl-6 Negative: CD5, CD43 EBER: Negative

## Abbreviations

EBV: Epstein–Barr virus
EBER: EBV-encoded mRNA
ENKTCL: Extranodal NK/T-cell lymphoma
NK: Natural killer
TCR: T-cell receptor.
